# YIPF2 promotes chemotherapeutic agent-mediated apoptosis via enhancing TNFRSF10B recycling to plasma membrane in non-small cell lung cancer cells

**DOI:** 10.1038/s41419-020-2436-x

**Published:** 2020-04-17

**Authors:** Yingying Wang, Sen Guo, Dongmei Li, Yongkang Tang, Lei Li, Ling Su, Xiangguo Liu

**Affiliations:** Shandong Provincial Key Laboratory of Animal Cell and Developmental Biology, School of Life Sciences, Shandong University, Qingdao, China

**Keywords:** Non-small-cell lung cancer, Apoptosis

## Abstract

Non-small cell lung cancer (NSCLC) is the most common histological type of lung cancer, and the identification of the apoptotic process of NSCLC is vital for its treatment. Usually, both the expression level and the cell surface level of TNFRSF10B (TNF Receptor superfamily member 10B) will increase after treatment with some chemotherapeutic agents, which plays a critical role in the apoptosis induction. However, the exact molecular mechanism underlying TNFRSF10B regulation remains largely elusive. Here, we found that TNFRSF10B, along with a vesicular trafficking regulator protein, YIPF2, were upregulated after treatment with pemetrexed (PEM) in NSCLC cells. Besides, YIPF2 increased the surface level of TNFRF10B, while YIPF2 knockdown inhibited the upregulation of TNFRSF10B and its recycling to plasma membrane. In addition, RAB8 decreased the cell surface TNFRSF10B by promoting its removing from plasma membrane to cytoplasm. Furthermore, we found that YIPF2, RAB8 and TNFRSF10B proteins interacted physically with each other. YIPF2 could further inhibit the physical interaction between TNFRSF10B and RAB8, thereby suppressing the removing of TNFRSF10B from plasma membrane to cytoplasm mediated by RAB8 and maintaining its high level on cell surface. Finally, using bioinformatics database, the YIPF2-TNFRSF10B axis was confirmed to be associated with the malignant progression of lung cancer. Taken together, we show that YIPF2 promotes chemotherapeutic agent-mediated apoptosis via enhancing TNFRSF10B recycling to plasma membrane in NSCLC cells. These findings may be beneficial for the development of potential prognostic markers of NSCLC and may provide effective treatment strategy.

## Introduction

Nearly 85% of primary lung cancers are of the non-small-cell lung cancer (NSCLC) type worldwide, and most patients present with advanced or metastatic disease at diagnosis^1^. Apoptotic-associated receptor TNFRSF10B (also known as DR5 or TRAIL-R2) has been reported to play an important role in the apoptosis of various cancer cells^2–4^. It locates at the cell surface and is activated upon binding to its ligand TRAIL (tumor necrosis factor-related apoptosis inducing ligand) or aggregation induced by some agonistic antibodies such as Apomab, Tigatuzumab and TRA-8^5–8^. In addition, the activation or overexpression of TNFRSF10B will signal apoptosis through CASP8-mediated activation of caspase cascades^9^. The elevated expression level and the cell surface level of TNFRSF10B induced by some chemotherapeutic agents play a critical role during the apoptosis of tumor cells perhaps in a ligand-independent manner. Our previous study indicated that pemetrexed (PEM), an inhibitor of folic acid synthesis used for the treatment of NSCLC patients currently^10,11^, can induce apoptosis by upregulating the expression of TNFRSF10B in NSCLC cells^12^. Therefore, as an important mediator of the extrinsic apoptotic signaling pathway, TNFRSF10B has been attracted much more attention on cancer therapy^13–15^. Currently, most of the studies around the molecular mechanism underlying TNFRSF10B regulation were focused on the transcriptional regulation^16,17^. Reports indicate that proteins involved in endoplasmic reticulum stress, such as DDIT3, ATF4, TP53 and ATF3, can modulate TNFRSF10B expression at the transcriptional level^4,18,19^. Considering as a membrane receptor, here we propose that the vesicle transport of TNFRSF10B from the cytoplasm to plasma membrane should also be critical for its function.

YIPF2 is a member of YIP family whose name refers to the Ypt (yeast RAB GTPase)-interacting protein^20–22^. The YIP family proteins are predicted to have five transmembrane segments with an N-terminal exposed to the cytoplasm and a short C-terminal exposed to the lumen of the secretory pathway^22^. However, the detailed function of YIPF2 is still unclear due to limited research. YIPF2 has been reported to mainly locate in the trans-Golgi network (TGN) co-localized with virous RAB proteins, suggesting that it is potentially involved in vesicle transport^23,24^. Besides, YIPF2 can serve as the GDF (GDI-displacement factor) of RAB5/RAB22A, thus catalyzing the dissociation of RAB-GDI complexes and regulating CD147 endocytic recycling finally in hepatocellular carcinoma cells. In addition, RAB8, one member of RAB small G protein family, plays a role in membrane traffic between the TGN and the basolateral plasma membrane in many cells^25,26^. And the depletion of RAB8 inhibits the transport of transferrin (Tf) and Tf receptor (TfR) to the endocytic recycling compartment (ERC), thereby regulating the recovery of the TfR in fibrosarcoma cells^27,28^.

In this study, we attempt to explore the mechanism of chemotherapeutic agents-induced upregulation of TNFRSF10B and apoptosis in NSCLC cells, which will be beneficial for identifying the potential prognostic marker of NSCLC and developing effective treatment strategy.

## Results

### PEM induces YIPF2 upregulation and apoptosis in NSCLC cells

**Fig. 1:**
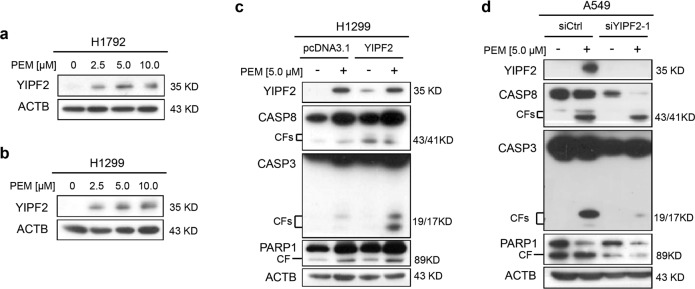
PEM induces YIPF2 upregulation and apoptosis in NSCLC cells. **a**, **b** H1792 (**a**) and H1299 (**b**) NSCLC cells were treated with PEM at various concentrations (0–10.0 μM) for 36 h. Cell lysates were analyzed by Western blotting with antibodies against YIPF2 and ACTB. **c** Overexpression of YIPF2 in H1299 cells in the presence or absence of PEM at 5.0 μM for 36 h. Cell lysates were analyzed by Western blotting with antibodies against YIPF2, CASP8, CASP3, PARP1 and ACTB. **d** Knockdown of YIPF2 expression by YIPF2–1 siRNA in A549 NSCLC cells in the presence or absence of PEM at 5.0 μM for 36 h. Cell lysates were analyzed by Western blotting with antibodies against YIPF2, CASP8, CASP3, PARP1 and ACTB.

In clinical practice, PEM has become a preferential drug for patients with NSCLC, which can induce apoptosis of cells^10,12,29^. Here, we treated the NSCLC cell lines H1792 and H1299 with PEM at different concentrations. Western blot assays showed that YIPF2, a protein which is potentially associated with vesicular transport, was markedly increased after the treatment with PEM (Fig. 1a, b). Besides, as shown in Fig. 1c, overexpression of YIPF2 in H1299 cells increased the levels of cleaved CASP8, CASP3 and PARP1, which were markers of extrinsic apoptosis pathway induced by PEM. In contrast, knockdown of YIPF2 expression in A549 cells decreased cleavage of the above proteins induced by PEM (Fig. 1d). Taken together, these data indicate that PEM upregulates levels of YIPF2 in NSCLC cells, and YIPF2 can further enhance PEM-induced apoptosis.

### PEM promotes apoptosis of NSCLC cells via YIPF2-TNFRSF10B axis

**Fig. 2:**
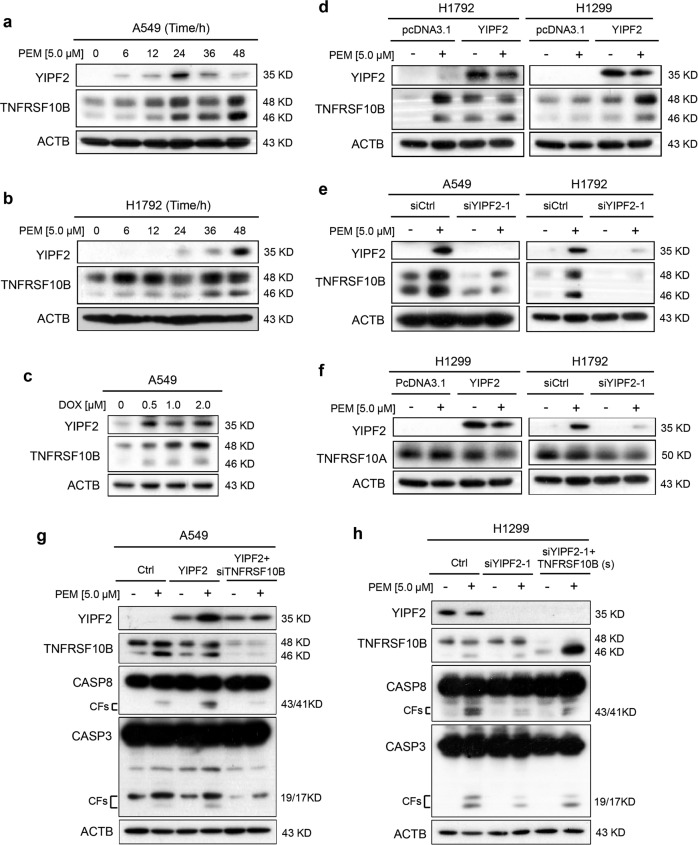
PEM induces apoptosis of NSCLC cells via YIPF2-TNFRSF10B axis. **a**, **b** A549 (**a**) and H1792 (**b**) cells were treated with PEM at 5.0 μM for the indicated times (0, 6, 12, 24, 36 and 48 h). Cell lysates were analyzed by Western blotting with antibodies against YIPF2, TNFRSF10B and ACTB. **c** A549 cells were treated with doxorubicin (DOX) at various concentrations (0–2.0 μM) for 18 h. Cell lysates were analyzed by Western blotting with antibodies against YIPF2, TNFRSF10B and ACTB. **d** Overexpression of YIPF2 in H1792 and H1299 cells in the presence or absence of PEM at 5.0 μM for 36 h. Cell lysates were analyzed by Western blotting with antibodies against YIPF2, TNFRSF10B and ACTB. **e** Knockdown of YIPF2 expression by YIPF2–1 siRNA in A549 and H1792 cells in the presence or absence of PEM at 5.0 μM for 36 h. Cell lysates were analyzed by Western blotting with antibodies against YIPF2, TNFRSF10B and ACTB. **f** Overexpression of YIPF2 in H1299 cells (left) or Knockdown of YIPF2 expression by YIPF2–1 siRNA in H1792 cells (right) in the presence or absence of PEM at 5.0 μM for 36 h. Cell lysates were analyzed by Western blotting with antibodies against YIPF2, TNFRSF10A and ACTB. **g** Three A549 cell lines (Ctrl, YIPF2, YIPF2 + siTNFRSF10B) in the presence or absence of PEM at 5.0 μM for 36 h. Cell lysates were analyzed by Western blotting with antibodies against YIPF2, TNFRSF10B, CASP8, CASP3 and ACTB. **h** Three H1299 cell lines (Ctrl, siYIPF2–1, siYIPF2–1 + TNFRSF10B (short isoform)) in the presence or absence of PEM at 5.0 μM for 36 h. Cell lysates were analyzed by Western blotting with antibodies against YIPF2, TNFRSF10B, CASP8, CASP3 and ACTB.

It has been reported that YIPF2 and its family members are potentially involved in intracellular vesicular transport, and knockdown of its expression promotes the migration of hepatocellular carcinoma cells^23,30^. However, it has not been identified whether YIPF2 mediates apoptosis in NSCLC cells. Here, A549 and H1792 cells were treated with 5.0 μM PEM for various times. Western blot analysis revealed that the expression of TNFRSF10B was also markedly increased compared with the controls, similar to the change of YIPF2 (Fig. 2a, b). In addition, another chemotherapeutic agent doxorubicin (DOX) was also found to upregulate the expression of YIPF2 and TNFRSF10B by western blot assays (Fig. 2c). As shown in Fig. 2d, e, western blot analysis revealed that levels of TNFRSF10B were significantly increased in YIPF2-overexpressed H1792 and H1299 cells compared with the controls, whereas knockdown of YIPF2 expression in A549 and H1792 cells led to opposite effects. However, PEM treatment or changing YIPF2 levels did not affect the expression of TNFRSF10A, which was TNF receptor superfamily member 10A (Fig. 2f). In addition, rescue experiment assays indicated that PEM-induced cleavages of CASP8 and CASP3 were greatly increased after overexpression of YIPF2 in A549 cells compared with that in control cells, whereas knockdown of TNFRSF10B expression in these cells simultaneously decreased the effects of PEM-induced cleavages of CASP8 and CASP3 (Fig. 2g). On the contrary, PEM-induced cleavages of CASP8 and CASP3 were greatly decreased after knockdown of YIPF2 expression in H1299 cells compared with the control cells, whereas overexpression of TNFRSF10B in these cells simultaneously increased the effects (Fig. 2h). These data above suggest that PEM induces apoptosis of NSCLC cells via YIPF2-TNFRSF10B axis.

### YIPF2 enhances TNFRSF10B recycling to plasma membrane

**Fig. 3:**
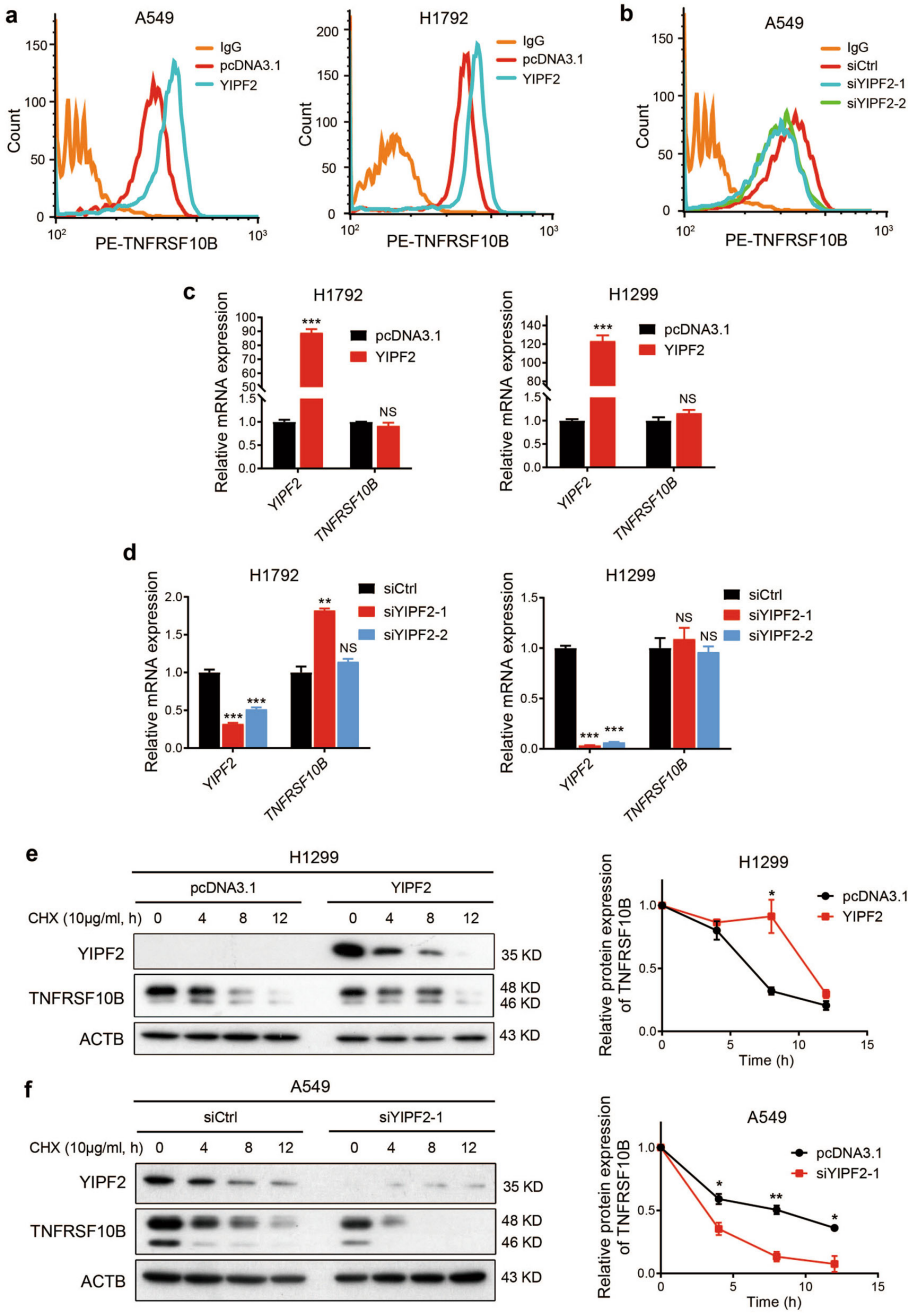
YIPF2 enhances TNFRSF10B recycling to plasma membrane. **a** Overexpression of YIPF2 in A549 and H1792 cells. The surface expression of TNFRSF10B was confirmed by flow cytometry analyses. **b** Knockdown of YIPF2 expression by YIPF2–1 and YIPF2–2 siRNA in A549 cells. The surface expression of TNFRSF10B was confirmed by flow cytometry analyses. **c** Relative RT-qPCR analyses of *YIPF2* and *TNFRSF10B* mRNA levels after YIPF2 overexpression in H1792 (left) and H1299 (right) cells (*n* = 3). **d** Relative RT-qPCR analyses of *YIPF2* and *TNFRSF10B* mRNA levels after YIPF2 knocking down in H1792 (left) and H1299 (right) cells (*n* = 3). **e** Left: Overexpression of YIPF2 in H1299 cells in the presence or absence of cycloheximide (CHX) at 10 μg/ml for the indicated times (0, 4, 8 and 12 h). Cell lysates were analyzed by Western blotting with antibodies against YIPF2, TNFRSF10B and ACTB. Right: The band intensity of TNFRSF10B was quantified by ImageJ software and plotted. This experiment was repeated three times independently with similar results. **f** Left: Knockdown of YIPF2 expression by YIPF2–1 siRNA in A549 cells in the presence or absence of cycloheximide (CHX) at 10 μg/ml for the indicated times (0, 4, 8 and 12 h). Cell lysates were analyzed by Western blotting with antibodies against YIPF2, TNFRSF10B and ACTB. Right: The band intensity of TNFRSF10B was quantified by ImageJ software and plotted. This experiment was repeated three times independently with similar results. (mean ± SEM, *n* = 3 independent experiments; NS, not significant; *P < 0.05, **P < 0.01 and ***P < 0.001; *P*-values in **c**, **e** and **f** were obtained using two-tailed Student’s *t*-tests, *P*-values in **d** were obtained using one-way ANOVA followed by Bonferroni’s post-hoc test).

It is well known that TNFRSF10B is mainly localized on the plasma membrane and acts as an apoptotic receptor to induce cell apoptosis. In order to investigate how YIPF2 affects the homeostasis of TNFRSF10B, we overexpressed YIPF2 in A549 and H1792 cells. The flow cytometry analysis showed that the levels of TNFRSF10B on the plasma membrane were upregulated compared with the control treatment (Fig. 3a). Besides, the cell surface expression of TNFRSF10B was markedly decreased after knockdown of YIPF2 in A549 cells (Fig. 3b). The RT-qPCR analysis revealed that overexpression of YIPF2 in H1792 and H1299 cells did not substantially alter the mRNA levels of *TNFRSF10B* (Fig. 3c). Similarly, knockdown of YIPF2 expression in the above two cells still did not change the mRNA levels of *TNFRSF10B* (Fig. 3d). Next, H1299 cells were treated with 10 μg/ml cycloheximide (CHX) for various times to inhibit new protein translation and examine the turnover of TNFRSF10B protein. Figure 3e showed increased stability of TNFRSF10B protein after YIPF2 overexpression compared with controls in H1299 cells, whereas Fig. 3f revealed stability of TNFRSF10B protein was decreased after YIPF2 knockdown compared with controls in A549 cells. These results were further confirmed by quantitative analysis (Fig. 3e, f). Altogether, these data suggest that YIPF2 enhances TNFRSF10B recycling to plasma membrane.

### RAB8 suppresses PEM-induced apoptosis of NSCLC cells by promoting the removing of TNFRSF10B from plasma membrane to cytoplasm

**Fig. 4:**
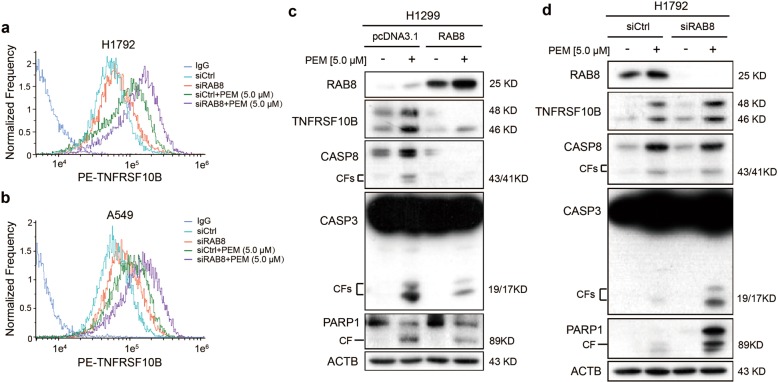
RAB8 suppresses PEM-induced apoptosis of NSCLC cells by promoting the removing of TNFRSF10B from plasma membrane to cytoplasm. **a**, **b** Knockdown of RAB8 expression by RAB8 siRNA in H1792 (**a**) and A549 (**b**) cells in the presence or absence of PEM at 5.0 μM for 36 h. The surface expression of TNFRSF10B was confirmed by flow cytometry analyses. **c** Overexpression of RAB8 in H1299 cells in the presence or absence of PEM at 5.0 μM for 36 h. Cell lysates were analyzed by Western blotting with antibodies against RAB8, TNFRSF10B, CASP8, CASP3, PARP1 and ACTB. **d** Knockdown of RAB8 expression by RAB8 siRNA in H1792 cells in the presence or absence of PEM at 5.0 μM for 36 h. Cell lysates were analyzed by Western blotting with antibodies against RAB8, TNFRSF10B, CASP8, CASP3, PARP1 and ACTB.

RAB8, one member of RAB small G protein family, has been reported to mediate protein recovery from the plasma membrane to the cytosol^27,28^. Interestingly, the surface level of TNFRSF10B was increased in RAB8 knocked-down H1792 and A549 cells compared with cells of control as detected by flow cytometry (Fig. 4a, b). Similarly, the level of TNFRSF10B on plasma membrane was also increased after the treatment of PEM (Fig. 4a, b). Furthermore, western blot assays showed that overexpression of RAB8 in H1299 cells decreased the level of TNFRSF10B and cleaved CASP8, CASP3 and PARP1, which were markers of extrinsic apoptosis pathway induced by PEM (Fig. 4c). In contrast, knockdown of RAB8 in H1792 cells increased the level of TNFRSF10B and the cleavage of CASP8, CASP3 and PARP1 induced by PEM (Fig. 4d). In summary, these results demonstrate that RAB8 inhibits PEM-induced apoptosis of NSCLC cells by promoting the removing of TNFRSF10B from plasma membrane to cytoplasm.

### YIPF2 inhibits the interaction between TNFRSF10B and RAB8

**Fig. 5:**
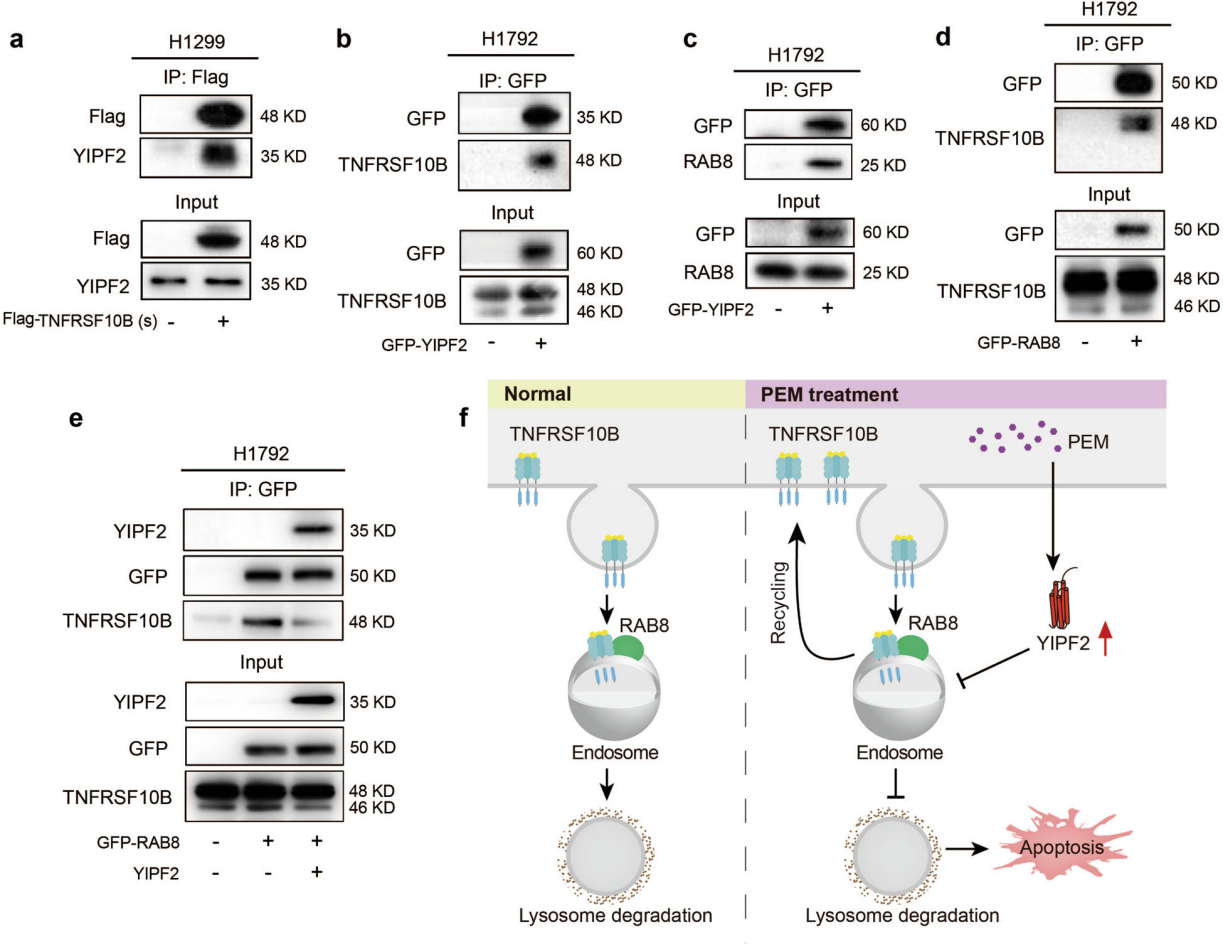
YIPF2 inhibits the interaction between TNFRSF10B and RAB8. **a** H1299 cells were transfected with pcDNA3.1 or pcDNA3.1-Flag-TNFRSF10B (short isoform) plasmids. Then the co-IP assays were carried out with Flag antibody and the co-eluted proteins were detected by western blot assays with Flag and YIPF2 antibodies. **b**, **c** H1792 cells were transfected with pEGFP-N1 or pEGFP-N1-YIPF2 plasmids. Then the co-IP assays were carried out with GFP antibody and the co-eluted proteins were detected by western blot assays with GFP, TNFRSF10B (**b**) and RAB8 (**c**) antibodies. **d** H1792 cells were transfected with pEGFP-N1 or pEGFP-N1-RAB8 plasmids. Then the co-IP assays were carried out with GFP antibody and the co-eluted proteins were detected by western blot assays with GFP, and TNFRSF10B antibodies. **e** Cell lysates were prepared from H1792 cells transiently co-expressing pEGFP-N1-RAB8 and pcDNA3.1-YIPF2. The co-IP assays were carried out with GFP antibody and the co-eluted proteins were detected by western blot assays with YIPF2, GFP and TNFRSF10B antibodies. **f** Schematic illustration showing that YIPF2 promotes PEM-mediated apoptosis via enhancing TNFRSF10B recycling to plasma membrane in NSCLC cells.

YIPF2 belongs to the YIP protein family, which has been reported to bind to RAB proteins to regulate vesicular transport^22,31^. Thus, we performed co-immunoprecipitation (co-IP) assays to investigate why YIPF2 increased the surface expression of TNFRSF10B. Briefly, H1299 cells were transfected with pcDNA3.1 or pcDNA3.1-Flag-TNFRSF10B (short isoform) plasmids. The co-IP assays revealed that TNFRSF10B could certainly interact with endogenous YIPF2 physically (Fig. 5a). In addition, YIPF2 could also interact with endogenous TNFRSF10B in H1792 cells transfected with pEGFP-N1 or pEGFP-N1-YIPF2 plasmids (Fig. 5b). Using similar methods, we further verified the interaction between YIPF2 and endogenous RAB8 in H1792 cells (Fig. 5c). The interaction between RAB8 and endogenous TNFRSF10B was also confirmed in H1792 cells by co-IP assays (Fig. 5d). Finally, RAB8 and YIPF2 were overexpressed in H1792 cells, and co-IP assays revealed that YIPF2 reduced the interaction between RAB8 and endogenous TNFRSF10B in H1792 cells compared with that in control cells (Fig. 5e). Taken together, these results suggest that YIPF2, RAB8 and TNFRSF10B proteins interact with each other, and YIPF2 inhibits the interaction between TNFRSF10B and RAB8, thereby suppressing the removing of TNFRSF10B from plasma membrane to cytoplasm through RAB8 and maintaining its high level on plasma membrane.

### YIPF2 and TNFRSF10B are associated with malignant progression in lung cancer patients

**Fig. 6:**
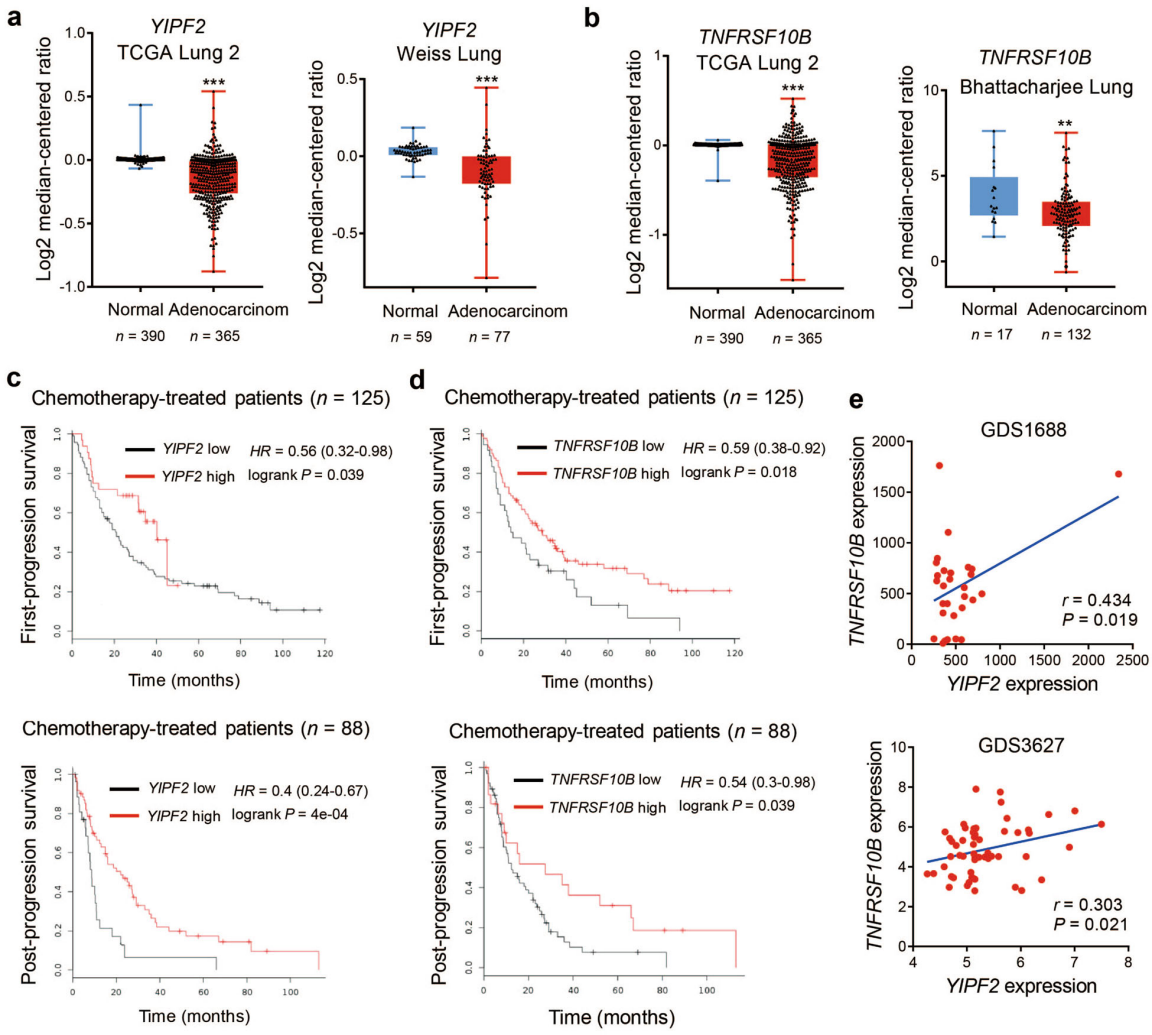
YIPF2 and TNFRSF10B are associated with malignant progression in lung cancer patients. **a** Box plots of *YIPF2* mRNA levels determined from two Oncomine datasets, namely TCGA Lung 2 and Weiss Lung (**P < 0.01 and ***P < 0.001; *P-*values were obtained using two-tailed Student’s *t*-tests). **b** Box plots of *TNFRSF10B* mRNA levels determined from two Oncomine datasets, namely TCGA Lung 2 and Bhattacharjee Lung. **c** Kaplan-Meier plots of the first-progression survival (FPS, upper) and post-progression survival (PPS, lower) of chemotherapy-treated patients stratified by *YIPF2* expression. The data were acquired from the Kaplan-Meier plotter database (*P*-values were obtained using the log-rank test). **d** Kaplan-Meier plots of the first-progression survival (FPS, upper) and post-progression survival (PPS, lower) of chemotherapy-treated patients stratified by *TNFRSF10B* expression. The data were acquired from the Kaplan-Meier plotter database (*P*-values were obtained using the log-rank test). **e** Scatter plots showing the correlation of *YIPF2* expression with *TNFRSF10B* expression in lung cancer cells in two GEO datasets (upper: GDS1688 which contains 29 lung cancer cell lines; lower: GDS3627 which contains 58 NSCLC cell lines). The *r* value was calculated via Spearman’s rank correlation coefficient analysis.

To determine whether our findings are clinically relevant, two Oncomine datasets, namely TCGA Lung 2 and Weiss Lung, were used to examine the mRNA expression of *YIPF2* (Fig. 6a). The data showed that the mRNA levels of *YIPF2* were significantly lower in lung adenocarcinoma tissues than that in normal tissues. Similarly, mRNA expression of *TNFRSF10B* was also lower in lung adenocarcinoma tissues than that in normal tissues in two Oncomine datasets (TCGA Lung 2 and Bhattacharjee Lung) (Fig. 6b). Using the Kaplan-Meier method followed by the log-rank test, we further confirmed that higher expression of *YIPF2* was correlated with higher first-progression survival (FPS, upper) and post-progression survival (PPS, lower) in chemotherapy-treated patients (Fig. 6c). Similarly, higher TNFRSF10B mRNA levels were also correlated with higher first-progression survival (FPS, upper) and post-progression survival (PPS, lower) in chemotherapy-treated patients (Fig. 6d). Finally, *YIPF2* expression tended to be positively associated with the expression of *TNFRSF10B* in two GEO datasets (GDS1688 and GDS3627), which contained 29 lung cancer cell lines and 58 NSCLC cell lines respectively (Fig. 6e). Collectively, these data reveal that the mRNA expression of *YIPF2* and *TNFRSF10B* is associated with malignant progression in lung cancer patients.

## Discussion

Currently, there are many studies focusing on the proliferation and apoptosis of NSCLC cells, aiming to obtain more effective treatments^32^. Randomized trials show that PEM has a good therapeutic effect and has become a preferential drug for patients with NSCLC^33,34^. Three enzymes used in purine and pyrimidine synthesis will be blocked by PEM, which are thymidylate synthase (TS), dihydrofolate reductase (DHFR) and glycinamide ribonucleotide formyltransferase (GARFT)^35^. Thus, PEM treatment inhibits the cellular DNA machinery via disruption of folic acid metabolism, thereby preventing cellular division and replication and causing cell cycle arrest and apoptosis^12,29,36^.

In the study, we found that the expression of YIPF2 was increased after PEM treatment, and its overexpression could further promote PEM-induced apoptosis in NSCLC cells. YIPF2 belongs to YIP family which has been reported to interact with RAB small G protein and plays vital roles in intracellular vesicular transport^20,22,24,31,37^. Numerous reports have discovered that some chemotherapeutic agents including PEM can induce endoplasmic reticulum (ER) stress in tumor cells^12,38^. We found that the expression of YIPF2, along with a hallmark transcription factor of ER stress, XBP1S, were upregulated after treatment with ER stress-inducing agents, thapsigargin (TG) and tunicamycin (TM) in NSCLC cells (data not shown). Furthermore, YIPF2 was conformed to be the target gene of XBP1S which binds to the promoter region of *YIPF2* (data not shown), suggesting that chemotherapeutic agents mediated upregulation of YIPF2 potentially via ER stress pathway. This project requires further experimental verification in the future.

A key finding of our study is that the elevated YIPF2 maintains high levels of TNFRSF10B on cell surface via inhibiting its removing from plasma membrane to cytoplasm mediated by RAB8. It is well known that two major apoptotic signaling pathways exist in cells: the intrinsic mitochondria-mediated pathway and the extrinsic death receptor-induced pathway, and TNFRSF10B is an important mediator of the extrinsic apoptotic signaling pathway^39^. As a membrane receptor, vesicle transport of TNFRSF10B between cytoplasm and plasma membrane is critical for its function and should be paid more attention. However, there are rare researches about the vesicle transport of TNFRSF10B in the literature. It has been reported that the translocation of TNFRSF10B from TGN to plasma membrane is regulated by cargo transport proteins, such as ARF1, RHO GAP protein ARAP1^40^ and nuclear translocation signaling proteins^41^. In addition, the faulty trafficking of TNFRS10B to plasma membrane will lead to its deficiency on cell surface and accumulation in cytosol^41^, TGN^40^ and nuclear perimeter^42^. On the contrary, the internalization of TNFRSF10B will occur upon binding to its ligand TRAIL, initiating extrinsic death receptor-induced apoptotic pathway. It has been reported that the endocytosed TNFRSF10B will be trafficked to the lysosome by RAB7 for degradation^43^. As a result, lysosomal degradation prevents receptor recycling back to the plasma membrane for further TRAIL ligation.

We also found that the surface level of TNFRSF10B was increased in RAB8 knockdown NSCLC cells, and RAB8 interacted with TNFRSF10B and YIPF2, suggesting that RAB8 participates in membrane trafficking of TNFRSF10B potentially through promoting its removing from plasma membrane to cytoplasm. Furthermore, co-IP assays confirmed that YIPF2-induced upregulation of TNFRSF10B on cell surface is due to inhibition of the interaction between RAB8 and TNFRSF10B. Besides, using Kaplan-Meier plotter database, *RAB8* mRNA levels were found to be correlated with survival rates in lung cancer patients (data not shown). As a GTPase, the activity of RAB8 is regulated by guanine nucleotide exchange factors (GEFs) that mediate GTP loading, also by GTPase activating proteins (GAPs) that convert them into inactive GDP-forms. RAB8-specific GEFs contains Rabin8 and GRAB^44–46^. Endogenous RAB8 is found in dynamic cell structures like filopodia, lamellipodia, protrusions, ruffles, and primary cilia^26^. In addition, RAB8 is involved in many important processes of cells such as cell migration^47^, neuron differentiation^27^, ciliogenesis^48^, epithelial polarization^49^, especially in membrane trafficking^27,50^. The function of RAB8 to participate in membrane trafficking is exactly what our research focuses on. Endogenous RAB8 is found to associate with vesicles, macropinosomes, and tubular structures via recognition by RAB8-specific antibody^27,44^. Furthermore, previous studies showed that RAB8 colocalizes with ARF6, EHD1, EHD3, ITGB1, MHCI, MYO5B, and MYO5C^27,51–53^, indicating that it participates in a recycling pathway based on clathrin-independent endocytosis. Consistently with TNFRSF10B, it has been described that Rab8 promotes the relocalization of Huntingtin protein to vesicles^54^.

In summary, we found that the expression of YIPF2 is increased after PEM treatment, which could promote the recycling of TNFRSF10B to plasma membrane by inhibiting the interaction between TNFRSF10B and RAB8, thus enhancing apoptosis of NSCLC cells eventually (Fig. 5f). Our findings provide a novel insight into the mechanism underlying TNFRSF10B regulation and will be beneficial for identifying the potential prognostic marker for NSCLC treatment.

## Materials and methods

### Cell lines and cell culture

The human NSCLC cell lines H1792, H1299 and A549 were originally obtained from the American Type Culture Collection (ATCC). They were cultured in RPMI 1640 medium with 10% FBS at 37 °C in a humidified atmosphere consisting of 5% CO2 and tested for no mycoplasma contamination.

### Reagents and antibodies

PEM was purchased from Sigma-Aldrich. CHX was purchased from MedChem Express. The TNFRSF10B antibody used in flow cytometry was obtained from Thermo Fisher Scientific (Cat. no. 12-9908-42; eBioscience). The primary antibodies used in western blot assays and Immunoprecipitation were as follows: anti-YIPF2 (Cat. no. HPA019902; Sigma-Aldrich), ACTB (Cat. no. A1978; Sigma-Aldrich), CASP8 (Cat. no. 9746S; CST), CASP3 (Cat. no. NB100-56708; Novus Biologicals), PARP1 (Cat. no. 9542S; CST), TNFRSF10B (Cat. no. 2019; ProSci Incorporated), RAB8 (Cat. no. 6975S; CST), Flag (Cat. no. F7425, F1804; Sigma-Aldrich) and GFP (Cat. no. G1544, Sigma-Aldrich; Cat. no. sc-9996, Santa Cruz).

### siRNA and plasmid transfection

The cells were transfected with jetPRIME transfection reagent (Polyplus transfection) or LipoMax reagent (Sudgen Biotechnology) in serum-free Opti-MEM (Gibco) according to the instruction manual. All siRNAs were synthesized from GenePharma (Shanghai, China). The sense and anti-sense strands of siRNAs were as follows: YIPF2 siRNA-1 sense: 5′-GGCUGUAAGUUGUACUUCUTT-3′ YIPF2 siRNA-1 antisense: 5′ -AGAAGUACAACUUACAGCCTT-3′

YIPF2 siRNA-2 sense: 5′-GUGCCACGUUGGCCUUUGUTT-3′ YIPF2 siRNA-2 antisense: 5′-ACAAAGGCCAACGUGGCACTT-3′

TNFRSF10B siRNA sense: 5′-GACCCUUGUGCUCGUUGUCTT-3′ TNFRSF10B siRNA antisense: 5′-GACAACGAGCACAAGGGUCTT-3′

RAB8 siRNA sense: 5′- GAGAATTAAACTGCAGATATT-3′ RAB8 siRNA antisense: 5′- GCTCGATGGCAAGAGAATTTT-3′

YIPF2, TNFRSF10B and RAB8 coding regions were amplified from A549 cDNA and subcloned into pcDNA3.1 or pEGFP-N1 vector. The primers were as follows:

YIPF2 sense: 5′-CCCGCTCGAGGCCGCCACCATGGCATCGGCCGACGAGCTGACCTTCC-3′ YIPF2 antisense: 5′-CCGCGGATCCCGGGAGGGGGCCAGGGACTGCGGCAAG-3′

TNFRSF10B sense: 5′-CGGATCCGCCGCCACCATGGAACAACGGGGACAGAACGC-3′ TNFRSF10B antisense: 5′-CCTCGAGTTAATGGTGATGGTGATGATGGGACATGGCAGAGTCTGCAT-3′

RAB8 sense: 5′-CGGTACCGCCGCCACCATGGCGAAGACCTACGATTAC-3′ RAB8 antisense: 5′-CGGATCCCCTCACAGAAGAACACATCGG-3′

### Western blot analysis

Cells were harvested and rinsed with pre-chilled PBS on ice. They were lysed in lysis buffer on ice for 30 min and then purified via centrifugation for 13 min at 4 °C. Protein extracts were resolved through 8%–15% SDS-PAGE, transferred to PVDF membranes, and probed with primary antibodies. Peroxidase-conjugated anti-mouse or rabbit antibody (Bio-Rad Laboratories) was used as secondary antibody and the antigen-antibody reaction was visualized by enhanced chemiluminescence assay.

### Immunoprecipitation

Cells were lysed in lysis buffer (20 mM Tris-HCl, pH 7.5; 150 mM NaCl; 1 mM Na2EDTA; 1 mM EGTA; 2.5 mM sodium pyrophosphate; 1 mM β-glycerophosphate; 1 mM Na_3_VO_4_; 0.5% Triton) on ice for 30 min then purified via centrifugation for 15 min at 4 °C. The supernatants were incubated with antibody at 4 °C for 1 h. Then the mixture was incubated with protein A beads (ThermoFisher) at 4 °C for 2 h. The beads were washed twice with 1 ml of lysis buffer. 20 μl 2× SDS buffer were added for elution (100 °C, 10 min). Samples were centrifuged for western blot analysis.

### Flow cytometry

To assay the expression of TNFRSF10B on the plasma membrane, 10^6^ cells were incubated at room temperature for 30 min with 5 μl of nonspecifc isotype-matched control IgG and 5 μl of mouse monoclonal antibodies conjugated with PE fluorochrome (Cat. no. 12-9908-42; eBioscience). Unbound antibodies were removed by washing the cells twice in PBS buffer. Analysis was performed on a Guava EasyCyte flow cytometer (Merck Millipore), and data were processed and presented using FlowJo software.

### RT-qPCR

Reverse transcription–quantitative real-time PCR (RT-qPCR) was performed with a LightCycler 480 System (Roche Diagnostics), using Real-Time PCR Super Mix (Mei5Bio, China) according to the manufacturer’s instruction. All reactions were done in a 20 μl reaction volume in triplicate. Primers were obtained from Sangon Biotech. Following an initial denaturation at 95 °C for 30 s, 40 cycles of PCR amplification were performed at 95 °C for 5 s and 60 °C for 30 s. Standard curves were generated and the relative amount of target gene mRNA was normalized to GAPDH. The primer sequences are as following:

*YIPF2* forward: 5′-TTCGAGGAGGCCACTAATCTT-3′ *YIPF2* reverse: 5′-AGTAGCTGAAGGTCCAGAATCC-3′ *TNFRSF10B* forward: 5′-GCCCCACAACAAAAGAGGTC-3′ *TNFRSF10B* reverse: 5′-AGGTCATTCCAGTGAGTGCTA-3′

*GAPDH* forward: 5′-ACGGATTTGGTCGTATTGGG-3′ *GAPDH* reverse: 5′-CGCTCCTGGAAGATGGTGAT-3′

### Statistics

All statistical analyses were performed using SPSS for Windows version 13.0 (SPSS). Two-tailed Student’s *t*-tests were used for comparisons between two groups, and one-way ANOVA followed by Bonferroni’s post-hoc test was used for multiple comparisons (three or more groups). The Kaplan-Meier curves for survival analyses were determined using the log-rank test. Spearman’s rank correlation coefficient analysis was performed to assess the correlation of *YIPF2* expression with *TNFRSF10B* expression in GEO datasets. All experiments for cell cultures were performed independently at least three times and in triplicate each time. In all cases, *P*-values < 0.05 were considered statistically significant.
